# New results on reachable set bounding for linear time delay systems with polytopic uncertainties via novel inequalities

**DOI:** 10.1186/s13660-017-1552-3

**Published:** 2017-11-09

**Authors:** Hao Chen, Shouming Zhong

**Affiliations:** 1 School of Mathematical Sciences, Huaibei Normal University, Huaibei, 235000 China; 2 School of Mathematical Sciences, University of Electronic Science and Technology of China, Chengdu, 611731 China

**Keywords:** reachable set, polytopic uncertainties, Lyapunov-Krasovskii (L-K) functional, linear matrix inequality (LMI)

## Abstract

This work is further focused on analyzing a bound for a reachable set of linear uncertain systems with polytopic parameters. By means of L-K functional theory and novel inequalities, some new conditions which are expressed in the form of LMIs are derived. It should be noted that novel inequalities can improve upper bounds of Jensen inequalities, which yields less conservatism of systems. Consequently, some numerical examples demonstrate that the authors’ results are somewhat more effective and advantageous compared with the previous results.

## Introduction

It is well known that reachable set estimation was first researched in the late 1960s for state estimations. The reachable set is a hot issue since the time due to its important and wide application in the design of controller and aircraft collision avoidance and peak-to-peak gain minimization problems. The reachable set of dynamic differential systems with delay and disturbance is a set that contains all the reachable trajectories from origin by outside peak input values [1–4]. In the real world, as for dynamic systems, there are two phenomena that cannot be avoided: time delays and uncertainties [5–15]. In fact, delays and the coefficients of differential equations in modeling progresses are obtained only approximately [16–18]. There are already some relevant outstanding results about reachable set estimation of dynamic systems. However, we think it is necessary to obtain a more tighter bound for a reachable set.

As pointed out in [16–20], an equation of one class can be transformed to an equation belonging to the external form of the other class. Thus, it is natural to classify the equations according to the properties of operators generated by the equations. In this paper, uncertain polytopic delayed linear systems with disturbances will be studied.

All the results about reachable set bounding are in the term of linear matrix inequalities (LMIs). The authors give an ellipsoid condition of the reachable set for linear systems without any delay [5]. The authors Fridman and Shaked improved the model [21]. They studied the linear systems with varying delays with peak inputs and got LMIs conditions of an ellipsoid by using the Razumikhin theory. After that, Kim got a more exact condition by constructing the modified Lyapunov-Razumikhin functionals [22]. Combining the decomposition technique, Nam derived a modified reachable set bound [23]. Actually, the reachable set is not an ellipsoid and it is only a closed set. Zuo et al. gave a non-ellipsoidal bound of a reachable set for linear time-delayed systems by the means of the maximal Lyapunov functionals and the Razumikhin method [24].

It should be noted that discrete delay is varying $0\leq\tau(t) \leq\tau$ in most previous literature. That is, the lower bound of discrete delay is 0. In fact, $\tau(t)$ varying $\tau_{m}\leq\tau(t) \leq\tau_{M}$ may describe the delay more exactly. In order to control the behavior of the system better, we hope to propose tighter reachable set estimation. Park, Lee and Lee proposed some novel inequalities which can be used to estimate integrations [25]. Therefore, those inequalities can be employed to estimate some integration terms of Lyapunov functionals for dynamic systems. Motivated by the above mentioned discussions, we consider the linear time-varying delay systems with polytopic uncertainties. Using novel inequalities, we derive a modified reachable set bound for the linear system with discrete delay $\tau_{m}\leq\tau(t)\leq\tau_{M}$. Moreover, four examples are given to demonstrate the effectiveness and advantage of our results.

In this paper, the used notations are listed as follows. Real matrix $P>0$ (≥0) denotes that *P* is a symmetric positive definite matrix (positive semi-definite). Superscript ‘T’ is transposition of a vector and a matrix; ∗ means the elements below the main diagonal in a symmetric block matrix; *I* is an identity matrix; ‘−’ in tables means that there is no feasible solution for linear matrix inequalities.

## Preliminaries

Consider uncertain polytopic delayed linear systems with disturbances in the form

1 $$ \begin{gathered} \dot{z}(t)=(A+\Delta A)z(t)+(D+\Delta D)z\bigl(t- \tau(t)\bigr)+(B+\Delta B)w(t), \\ z(t)=0, \quad t\in[-\tau_{M},0], \end{gathered} $$

where $z(t)\in R^{n}$ is a state vector; $w(t)\in R^{m}$ is outside disturbance. $A, \Delta A\in R^{n\times n}$, $D, \Delta D\in R^{n \times n}$, $B, \Delta B\in R^{n\times m}$. *A*, *D*, *B* are known matrices. Δ*A*, Δ*D*, Δ*B* are uncertain matrices. $\tau(t)$ is time delay.

Discrete delay $\tau(t)$ and disturbance $w(t)$ are assumed to be as follows:

$$\begin{aligned}& \tau_{m}\leq\tau(t)\leq\tau_{M}, \quad\quad 0\leq\dot{ \tau}(t)\leq\mu< 1, \\& w^{T}(t)w(t)\leq w_{m}^{2}, \end{aligned}$$

where *μ*, $w_{m}$ are constant.

The uncertainty parameter matrices are expressed by a linear convex-hull of matrices $A_{i}$, $B_{i}$ and $D_{i}$

$$ \Delta A=\sum_{i=1}^{N} \theta_{i}(t)A_{i},\quad\quad \Delta B=\sum _{i=1} ^{N}\theta_{i}(t)B_{i}, \quad\quad \Delta D=\sum_{i=1}^{N} \theta_{i}(t)D _{i} $$

with $\theta_{i}(t)\in[0,1]$ and $\sum_{i=1}^{N}\theta_{i}(t)=1$, $\forall t>0$. $A_{i}$, $B_{i}$ and $D_{i}$ are known matrices.

In order to obtain reachable set bounds for a linear dynamic system, we state some useful lemmas and some novel inequalities firstly.

### Lemma 1

([26])

*As for the well*-*defined integral* $\int_{a(t)}^{b(t)}f(t,s)\,ds$, *the following relation known as the Leibniz rule holds*:

$$ \frac{d}{dt} \int_{a(t)}^{b(t)}f(t,s)\,ds=\dot{b}(t)f\bigl[t,b(t) \bigr]-\dot{a}(t)f\bigl[t,a(t)\bigr] + \int_{a(t)}^{b(t)}\frac{\partial}{\partial t}f(t,s)\,ds. $$

### Lemma 2

([25])

*For a positive definite matrix* $R>0$ *and a differentiable function* $\{z(u)| u\in[a,b]\}$, *the following inequalities hold*:

$$\begin{aligned}& (b-a) \int_{a}^{b}z^{T}(s)Rz (s)\,ds\geq \xi_{1}^{T}\left [ \begin{matrix} 4R & -6R \\ * & 12R \end{matrix} \right ] \xi_{1}, \\& (b-a) \int_{a}^{b}\dot{z}^{T}(s)R\dot{z}(s)\,ds \,d\theta\geq\xi_{2} ^{T}\left [ \begin{matrix} 9R & -3R & 24R & -60R \\ *& 9R& -36R & 60R \\ * & * & 192R & -360R \\ * & * & * & 720R \end{matrix} \right ] \xi_{2}, \\& \int_{a}^{b} \int_{\theta}^{b}\dot{z}^{T}(s)R\dot{z}(s)\,ds \,d\theta \geq\xi_{3}^{T}\left [ \begin{matrix} 6R & 6R & -24R \\ * & 18R & -48R \\ *& *& 144R \end{matrix} \right ] \xi_{3}, \end{aligned}$$

*where*

$$\begin{aligned}& \xi_{1}^{T} = \biggl(\frac{1}{b-a} \int_{a}^{b}z^{T}(s)\,ds, \frac{1}{(b-a)^{2}} \int_{a}^{b} \int_{\theta}^{b}z^{T}(s)\,ds\,d\theta\biggr), \\& \xi_{2}^{T} = \biggl(z^{T}(b),z^{T}(a), \frac{1}{b-a} \int_{a}^{b}z^{T}(s)\,ds, \frac{1}{(b-a)^{2}} \int_{a}^{b} \int_{\theta}^{b}z^{T}(s)\,ds\,d\theta\biggr), \\& \xi_{3}^{T} = \biggl(z^{T}(b),\frac{1}{b-a} \int_{a}^{b}z^{T}(s)\,ds, \frac{1}{(b-a)^{2}} \int_{a}^{b} \int_{\theta}^{b}z^{T}(s)\,ds\,d\theta\biggr). \end{aligned}$$

### Lemma 3

([5])

*Let* *V* *be a Lyapunov function for system* (1) *with* $w^{T}(t)w(t)\leq w_{m}^{2}$. *If*

$$ \dot{V}+\alpha V-\frac{\alpha}{w_{m}^{2}}w^{T}(t)w(t)\leq0, $$

*then* $V\leq1$.

## Main results

In this section, we will firstly consider a reachable set for uncertain parameter matrices $\Delta A=0$, $\Delta D=0$, $\Delta B=0$ in system (1), namely,

2 $$ \dot{z}(t)=Az(t)+Dz\bigl(t-\tau(t)\bigr)+Bw(t),\quad \quad z(t)=0, \quad t\in[-h,0]. $$

After that, we will consider a reachable set for uncertain system (1).

If $\tau_{m}\leq\tau(t)\leq\tau_{M}$, $\dot{\tau}(t)\leq\mu<1$, we get the reachable set bounding for dynamic system (2) in Theorem 1.

### Theorem 1

*If there exist appropriate dimension matrices* $P>0$, $R_{1}>0$, $R_{2}>0$, $Q _{1}>0$, $Q_{2}>0$, $M_{1}>0$, $M_{2}>0$, $M_{3}>0$, *appropriate dimensions matrices* $N_{1}$, $N_{2}$, *and a scalar* $\alpha>0$ *such that the following inequality holds*:

3 $$\begin{aligned} \begin{aligned}[b] \Phi&= \left [ \textstyle\begin{array}{@{}c@{\quad}c@{\quad}c@{\quad}c@{\quad}c@{\quad}c@{\quad }c@{\quad}c@{\quad}c@{\quad}c@{\quad}c@{\quad}c@{}} \Phi_{11} & \Phi_{12} & \Phi_{13} & 0 & \Phi_{15} & \Phi_{16} & 0 & \Phi _{18} & \Phi_{19} & 0 & \Phi_{1\ 11} & \Phi_{1\ 12} \\ * & \Phi_{22} & 0 & 0 & 0 & 0 & 0 & 0 & 0 & 0 & \Phi_{2\ 11} & \Phi_{2\ 12} \\ * & * & \Phi_{33} & \Phi_{34} & \Phi_{35} & 0 & \Phi_{37} & \Phi_{38} & 0 & \Phi_{3,10} & 0 & 0 \\ * & * & * & \Phi_{44} & 0 & 0 & \Phi_{47} & 0 & 0 & \Phi_{4,10} & 0 & 0 \\ * & * & * & * & \Phi_{55} & 0 & 0 & \Phi_{58} & 0 & 0 & 0 & 0 \\ * & * & * & * & * & \Phi_{66} & 0 & 0 & \Phi_{69} & 0 & 0 & 0 \\ * & * & * & * & * & * & \Phi_{77} & 0 & 0 & \Phi_{7,10} & 0 & 0 \\ * & * & * & * & * & * & * & \Phi_{88} & 0 & 0 & 0 & 0 \\ * & * & * & * & * & * & * & * & \Phi_{99} & 0 & 0 & 0 \\ * & * & * & * & * & * & * & * & * & \Phi_{10\ 10} & 0 & 0 \\ * & * & * & * & * & * & * & * & * & * & \Phi_{11\ 11} & \Phi_{11\ 12} \\ * & * & * & * & * & * & * & * & * & * & * & \Phi_{12\ 12} \end{array}\displaystyle \right ] \\ &\leq0, \end{aligned} \end{aligned}$$

*where*

$$\begin{aligned}& \begin{aligned} \Phi_{11}&=\alpha P+PA+A^{T}P+R_{1}+M_{1}- 9e^{-\alpha\tau_{m}}Q_{1} - 12e^{-\alpha\tau_{m}}M_{2} \\ &\quad{} - 12e^{-\alpha\tau_{M}}M_{3}+N_{2}A+A^{T}N_{2}^{T} +A^{T}N_{1}A+A ^{T}N^{T}_{1}A, \end{aligned} \\& \Phi_{12}=PD+N_{2}D+A^{T}N_{1}D+A^{T}N^{T}_{1}D, \quad\quad \Phi_{13}=3e ^{-\alpha\tau_{m}}Q_{1}, \\& \Phi_{15}=-24e^{-\alpha\tau_{m}}Q_{1}- 12e^{-\alpha\tau_{m}}M_{2},\quad\quad \Phi_{16}=- 12e^{-\alpha\tau_{M}}M_{3}, \\& \Phi_{18}=60e^{-\alpha \tau_{m}}Q_{1}+48e^{-\alpha\tau_{m}}M_{2}, \\& \Phi_{19}=48e^{-\alpha\tau_{M}}M_{3}, \quad\quad \Phi_{1\ 11}=-N_{2}-A ^{T}N^{T}_{1}-A^{T}N_{1}, \\& \Phi_{1\ 12}=PB+N_{2}B+A^{T}N_{1}B+A^{T}N^{T}_{1}B, \\& \Phi_{22}=-(1-\mu)e^{-\alpha\tau_{M}}M_{1}+D^{T}N_{1}D+D^{T}N^{T} _{1}D, \\& \Phi_{2\ 11}=-D^{T}N^{T}_{1}-D^{T}N_{1},\quad \quad\Phi_{2\ 12}=D^{T}N _{1}B+D^{T}N^{T}_{1}B, \\& \Phi_{33}=e^{-\alpha\tau_{m}}R_{2}-e^{-\alpha\tau_{m}}R_{1} -9e ^{-\alpha\tau_{m}}Q_{1}-9e^{-\alpha\tau_{M}}Q_{2}, \\& \Phi_{34}=3e^{-\alpha\tau_{M}}Q_{2}, \quad\quad \Phi_{35}=36e^{-\alpha \tau_{m}}Q_{1}, \quad \quad\Phi_{37}=-24e^{-\alpha\tau_{M}}Q_{2}, \quad \\& \Phi_{38}=-60e^{-\alpha\tau_{m}}Q_{1}, \quad\quad \Phi_{3 \ 10}=60e^{- \alpha\tau_{M}}Q_{2}, \\& \Phi_{44}=-e^{-\alpha\tau_{M}}R_{2}-9e^{-\alpha\tau_{M}}Q_{2},\quad \quad\Phi_{47}=36e^{-\alpha\tau_{M}}Q_{2},\quad\quad \Phi_{4\ 10}=-60e ^{-\alpha\tau_{M}}Q_{2}, \\& \Phi_{55}=-192e^{-\alpha\tau_{m}}Q_{1}- 36e^{-\alpha\tau_{m}}M _{2}, \quad\quad \Phi_{58}=360e^{-\alpha\tau_{m}}Q_{1}+96e^{-\alpha\tau _{m}}M_{2}, \\& \Phi_{6 6}=-36e^{-\alpha\tau_{M}}M_{3}, \quad\quad \Phi_{6 9}=96e^{- \alpha\tau_{M}}M_{3}, \\& \Phi_{77}=-192e^{-\alpha\tau_{M}}Q_{2}, \quad\quad \Phi_{7\ 10}=360e ^{-\alpha\tau_{M}}Q_{2}, \\& \Phi_{88}=-720e^{-\alpha\tau_{m}}Q_{1}- 288e^{-\alpha\tau_{m}}M _{2}, \\& \Phi_{9 9}=-288e^{-\alpha\tau_{M}}M_{3}, \quad\quad \Phi_{10\ 10}=-720e^{- \alpha\tau_{M}}Q_{2}, \\& \Phi_{11\ 11}=(\tau_{m})^{2}Q_{1}+( \tau_{M}-\tau_{m})^{2}Q_{2}+N _{1}+N^{T}_{1}, \quad \quad\Phi_{11\ 12}=-N_{1}B-N^{T}_{1}B, \\& \Phi_{12\ 12}=-\frac{\alpha}{w^{2}_{m}}+B^{T}N_{1}B+B^{T}N^{T}_{1}B. \end{aligned}$$

*Then the reachable sets of system* (2) *are bounded in a ball* $B(0,r)=\{z\in R^{n}|\Vert z\Vert \leq r\}$ *with*

4 $$ r=\frac{1}{\sqrt{\lambda_{\min}(P)}}. $$

### Proof

Construct the Lyapunov-Krasovskii functional

$$ V(z_{t})=\sum_{i=1}^{6}V_{i}(z_{t}), $$

where

$$\begin{aligned}& V_{1}(z_{t})=z^{T}(t)Pz(t), \\ & V_{2}(z_{t})= \int_{t-\tau_{m}}^{t}e^{\alpha(s-t)}z^{T}(s)R_{1}z(s) \,ds + \int_{t-\tau_{M}}^{t-\tau_{m}}e^{\alpha(s-t)}z^{T}(s)R_{2}z(s) \,ds, \\ & V_{3}(z_{t})= \int_{t-\tau(t)}^{t}e^{\alpha(s-t)}z^{T}(s)M_{1}z(s) \,ds, \\ & V_{4}(z_{t})=\tau_{m} \int_{-\tau_{m}}^{0} \int_{t+\theta}^{t}e^{ \alpha(s-t)} \dot{z}^{T}(s)Q_{1} \dot{z}(s)\,ds, \\ & V_{5}(z_{t})=(\tau_{M}-\tau_{m}) \int_{-\tau_{M}}^{-\tau_{m}} \int_{t+\theta}^{t} e^{\alpha(s-t)}\dot{z}^{T}(s)Q_{2} \dot{z}(s)\,ds, \\ & \begin{aligned} V_{6}(z_{t})&= \int_{t-\tau_{m}}^{t} \int_{\theta}^{t} \int_{\lambda} ^{t} e^{\alpha(s-t)} \dot{z}^{T}(s)M_{2}\dot{z}(s) \,ds\,d\theta\,d\lambda \\ & \quad{} + \int_{t-\tau_{M}}^{t} \int_{\theta}^{t} \int_{\lambda}^{t} e^{ \alpha(s-t)}\dot{z}^{T}(s)M_{3} \dot{z}(s)\,ds\,d\theta\,d\lambda. \end{aligned} \end{aligned}$$

Computing the derivative of $V(z_{t})$ of model (3), we have

5 $$\begin{aligned}& \begin{aligned}[b] \dot{V}_{1}(z_{t}) &=2z^{T}(t)P\dot{z}(t)=-\alpha V_{1}(z_{t})+2z^{T}(t)P \dot{z}(t)+\alpha z^{T}(t)Pz(t) \\ &=-\alpha V_{1}(z_{t})+\alpha z^{T}(t)Pz(t)+2z^{T}(t)P \bigl(Az(t)+Dz\bigl(t- \tau(t)\bigr)+Bw(t)\bigr), \end{aligned} \end{aligned}$$

6 $$\begin{aligned}& \begin{aligned}[b] \dot{V}_{2}(z_{t}) &= -\alpha V_{2}(z_{t})+z^{T}(t)R_{1}z(t)+e^{-\alpha \tau_{m}}z^{T}(t- \tau_{m})R_{2}z(t-\tau_{m}) \\ &\quad{} -e^{-\alpha\tau_{m}}z^{T}(t-\tau_{m})R_{1}z(t- \tau_{m}) -e^{- \alpha\tau_{M}}z^{T}(t-\tau_{M})R_{2}z(t- \tau_{M}), \end{aligned} \end{aligned}$$

7 $$\begin{aligned}& \begin{aligned}[b] \dot{V}_{3}(z_{t}) &= -\alpha V_{3}(z_{t})+z^{T}(t)M_{1}z(t)- \bigl(1- \dot{\tau}(t)\bigr)e^{-\alpha\tau(t)}z^{T}\bigl(t-\tau(t) \bigr)M_{1}z\bigl(t-\tau(t)\bigr) \\ &\leq-\alpha V_{3}(z_{t})+z^{T}(t)M_{1}z(t)- (1-\mu)e^{-\alpha\tau _{M}}z^{T}\bigl(t-\tau(t)\bigr)M_{1}z \bigl(t-\tau(t)\bigr), \end{aligned} \end{aligned}$$

8 $$\begin{aligned}& \begin{aligned}[b] \dot{V}_{4}(z_{t}) &= -\alpha V_{4}(z_{t})+\tau_{m}^{2} \dot{z}^{T}(t)Q _{1}\dot{z}(t) -\tau_{m} \int_{t-\tau_{m}}^{t}e^{\alpha(s-t)}\dot{z} ^{T}(s)Q_{1}\dot{z}(s)\,ds \\ &\leq-\alpha V_{4}(z_{t})+\tau_{m}^{2} \dot{z}^{T}(t)Q_{1}\dot{z}(t) -e^{-\alpha\tau_{m}} \tau_{m} \int_{t-\tau_{m}}^{t}e^{\alpha(s-t)} \dot{z}^{T}(s)Q_{1} \dot{z}(s)\,ds. \end{aligned} \end{aligned}$$

By using Lemma 3,

9 $$ \begin{aligned}[b] \dot{V}_{4}(z_{t}) &\leq-\alpha V_{4}(z_{t}) +\tau_{m}^{2} \dot{z}^{T}(t)Q _{1}\dot{z}(t) \\ &\quad{} -e^{-\alpha\tau_{m}}\zeta_{1}^{T}\left [ \begin{matrix} 9Q_{1} & -3Q_{1} & 24Q_{1} & -60Q_{1} \\ *& 9Q_{1}& -36Q_{1} & 60Q_{1} \\ * & * & 192Q_{1} & -360Q_{1} \\ * & * & * & 720Q_{1} \end{matrix} \right ] \zeta_{1}, \end{aligned} $$

where $\zeta_{1}^{T}=(z^{T}(t),z^{T}(t-\tau_{m}),\frac{1}{\tau_{m}} \int_{t-\tau_{m}}^{t}z^{T}(s)\,ds, \frac{1}{\tau_{m}^{2}}\int_{t-\tau _{m}}^{t}\int_{\theta}^{t}z^{T}(s)\,ds\,d\theta)$,

10 $$ \begin{aligned}[b] &\dot{V}_{5}(z_{t}) \\ &\quad = -\alpha V_{5}(z_{t})+(\tau_{M}- \tau_{m})^{2}\dot{z}^{T}(t)Q_{2} \dot{z}(t) -(\tau_{M}-\tau_{m}) \int_{t-\tau_{M}}^{t-\tau_{m}}e^{ \alpha(s-t)}\dot{z}^{T}(s)Q_{2} \dot{z}(s)\,ds \\ &\quad \leq -\alpha V_{5}(z_{t}) \\ &\quad\quad{} +(\tau_{M}-\tau_{m})^{2}\dot{z}^{T}(t)Q_{2} \dot{z}(t) -e^{-\alpha \tau_{M}}(\tau_{M}-\tau_{m}) \int_{t-\tau_{M}}^{t-\tau_{m}}e^{\alpha(s-t)} \dot{z}^{T}(s)Q_{2} \dot{z}(s)\,ds. \end{aligned} $$

By using Lemma 3,

11 $$ \begin{aligned}[b] \dot{V}_{5}(z_{t}) &\leq-\alpha V_{5}(z_{t}) +(\tau_{M}- \tau_{m})^{2} \dot{z}^{T}(t)Q_{2} \dot{z}(t) \\ &\quad{} -e^{-\alpha\tau_{M}}\zeta_{2}^{T}\left [ \begin{matrix} 9Q_{2} & -3Q_{2} & 24Q_{2} & -60Q_{2} \\ *& 9Q_{2}& -36Q_{2} & 60Q_{2} \\ * & * & 192Q_{2} & -360Q_{2} \\ * & * & * & 720Q_{2} \end{matrix} \right ] \zeta_{2}, \end{aligned} $$

where

12 $$\begin{aligned}& \begin{aligned} \zeta_{2}^{T} &= \biggl(z^{T}(t-\tau_{m}),z^{T}(t- \tau_{M}), \\ &\quad \frac{1}{\tau_{M}-\tau_{m}} \int_{t-\tau_{M}}^{t-\tau_{m}}z^{T}(s)\,ds, \frac{1}{(\tau_{M}-\tau_{m})^{2}} \int_{t-\tau_{M}}^{t-\tau_{m}} \int_{\theta}^{t-\tau_{m}}z^{T}(s)\,ds\,d\theta\biggr), \end{aligned} \\& \begin{aligned}[b] \dot{V}_{6}(z_{t}) &=-\alpha V_{6}(z_{t})+\frac{1}{2} \tau_{m}^{2} \dot{z}(t)M_{2}\dot{z}(t) + \frac{1}{2}\tau_{M}^{2}\dot{z}(t)M_{3} \dot{z}(t) \\ &\quad{} - \int_{t-\tau_{m}}^{t} \int_{\theta}^{t}e^{\alpha(s-t)}\dot{z}^{T}(s)M _{2}\dot{z}(s)\,ds\,d\theta- \int_{t-\tau_{M}}^{t} \int_{\theta}^{t}e^{ \alpha(s-t)}\dot{z}^{T}(s)M_{3} \dot{z}(s)\,ds\,d\theta \\ &\leq-\alpha V_{6}(z_{t})+\frac{1}{2} \tau_{m}^{2}\dot{z}(t)M_{2} \dot{z}(t) + \frac{1}{2}\tau_{M}^{2}\dot{z}(t)M_{3} \dot{z}(t) \\ &\quad{} -2e^{-\alpha\tau_{m}} \int_{t-\tau_{m}}^{t} \int_{\theta}^{t} \dot{z}^{T}(s)M_{2} \dot{z}(s)\,ds\,d\theta \\ &\quad{} -2e^{-\alpha\tau_{M}} \int_{t-\tau_{M}}^{t} \int_{\theta}^{t}\dot{z}^{T}(s)M_{3} \dot{z}(s)\,ds\,d \theta. \end{aligned} \end{aligned}$$

In the light of Lemma 3,

13 $$ \begin{aligned}[b] \dot{V}_{6}(z_{t}) &\leq-\alpha V_{6}(z_{t})+\frac{1}{2} \tau_{m}^{2} \dot{z}(t)M_{2}\dot{z}(t) + \frac{1}{2}\tau_{M}^{2}\dot{z}(t)M_{3} \dot{z}(t) \\ &\quad{} -2e^{-\alpha\tau_{m}} \zeta_{3}^{T}\left [ \begin{matrix} 6M_{2} & 6M_{2} & -24M_{2} \\ * & 18M_{2} & -48M_{2} \\ *& *& 144M_{2} \end{matrix} \right ] \zeta_{3} \\ &\quad{} -2e^{-\alpha\tau_{M}}\zeta_{4}^{T}\left [ \begin{matrix} 6M_{3} & 6M_{3} & -24M_{3} \\ * & 18M_{3} & -48M_{3} \\ *& *& 144M_{3} \end{matrix} \right ] \zeta_{4}, \end{aligned} $$

where

$$\begin{aligned}& \xi_{3}^{T}=\biggl(z^{T}(t), \frac{1}{\tau_{m}} \int_{t-\tau_{m}}^{t}z^{T}(s)\,ds, \frac{1}{\tau_{m}^{2}} \int_{t-\tau_{m}}^{t} \int_{\theta}^{t}z^{T}(s)\, ds\,d \theta \biggr), \\ & \xi_{4}^{T}=\biggl(z^{T}(t),\frac{1}{\tau_{M}} \int_{t-\tau_{M}}^{t}z^{T}(s)\, ds, \frac{1}{ \tau_{M}^{2}} \int_{t-\tau_{M}}^{t} \int_{\theta}^{t}z^{T}(s)\,ds\, d\theta \biggr). \end{aligned}$$

Certainly, the following equations hold:

14 $$ \begin{aligned} &2\bigl(-\dot{z}(t)+Az(t) \\ &\quad{}+ Dz\bigl(t-\tau(t)\bigr)+Bw(t)\bigr)^{T}N_{1} \bigl(- \dot{z}(t)+Az(t)+Dz\bigl(t-\tau(t)\bigr)+Bw(t)\bigr)=0, \\ &2z^{T}(t)N_{2}\bigl(-\dot{z}(t)+Az(t)+Dz\bigl(t-\tau(t) \bigr)+Bw(t)\bigr)=0, \end{aligned} $$

where $N_{1}$, $N_{2}$ are matrices with appropriate dimensions.

Through (5)-(7) and (9)(11)(13)(14), one gets

$$ \begin{aligned} &\dot{V}_{1}(z_{t})+ \dot{V}_{2}(z_{t})+\dot{V}_{3}(z_{t}) + \dot{V} _{4}(z_{t})+\dot{V}_{5}(z_{t})+ \dot{V}_{6}(z_{t}) -\frac{\alpha}{w _{m}^{2}}w^{T}(t)w(t) \\ &\quad \leq-\alpha V_{1}(z_{t})-\alpha V_{2}(z_{t})- \alpha V_{3}(z_{t}) - \alpha V_{4}(z_{t})- \alpha V_{5}(z_{t})-\alpha V_{6}(z_{t}) +\zeta ^{T}(t)\Phi\zeta(t). \end{aligned} $$

That is,

$$ \begin{aligned} \dot{V}(z_{t})-\frac{\alpha}{w_{m}^{2}}w^{T}(t)w(t) \leq-\alpha V(z _{t}) +\zeta^{T}(t)\Phi\zeta(t). \end{aligned} $$

Then one has

15 $$ \dot{V}(z_{t})+\alpha V(z_{t})- \frac{\alpha}{w_{m}^{2}}w^{T}(t)w(t) \leq\zeta^{T}(t)\Phi\zeta(t), $$

where

$$\begin{aligned} \zeta^{T}(t) &= \biggl[z^{T}(t), z^{T}\bigl(t-\tau(t)\bigr), z^{T}(t-\tau_{m}), z^{T}(t-\tau _{M}), \frac{1}{\tau_{m}} \int_{t-\tau_{m}}^{t}z^{T}(s)\,ds, \\ &\quad \frac{1}{\tau_{M}} \int_{t-\tau_{M}}^{t}z^{T}(s)\,ds, \frac{1}{\tau _{M}-\tau_{m}} \int_{t-\tau_{M}}^{t-\tau_{m}}z^{T}(s)\,ds, \frac{1}{ \tau_{m}^{2}} \int_{t-\tau_{m}}^{t} \int_{\theta}^{t}z^{T}(s)\,ds \, d\theta , \\ & \quad \frac{1}{\tau_{M}^{2}} \int_{t-\tau_{M}}^{t} \int_{\theta }^{t}z^{T}(s)\,ds \,d \theta, \frac{1}{(\tau_{M}-\tau_{m})^{2}} \int_{t-\tau_{M}}^{t-\tau _{m}} \int_{\theta}^{t-\tau_{m}}z^{T}(s)\,ds \,d\theta, \dot{z}^{T}(t), w^{T}(t)\biggr]. \end{aligned}$$

For (2) holding, we get $\dot{V}+\alpha V-\frac{\alpha}{w_{m}^{2}}w ^{T}(t)w(t)\leq0$.

Thus, according to Lemma 3, one has $V(z_{t})\leq1$.

It is easy to see

$$ z^{T}(t)Pz(t)=V_{1}(z_{t})\leq V_{1}(z_{t})+V_{2}(z_{t})+V_{3}(z_{t})+V _{4}(z_{t})+ V_{5}(z_{t})+V_{6}(z_{t}) = V(z_{t}). $$

Furthermore, by using the spectral property for symmetric positive definite matrix *P*, we get

16 $$ \lambda_{\min}(P)\bigl\Vert z(t)\bigr\Vert ^{2}\leq V(z_{t}). $$

Therefore, $\Vert z(t)\Vert \leq r=\frac{1}{\sqrt{\lambda _{\min}(P)}}$ due to (16). □

Next, let us consider the polytopic uncertain linear system (1). Reachable set bounding of system (1) is got and stated in Theorem 2.

### Theorem 2

*If there exist appropriate dimension matrices* $P>0$, $R_{1}>0$, $R_{2}>0$, $Q _{1}>0$, $Q_{2}>0$, $M_{1}>0$, $M_{2}>0$, $M_{3}>0$, *appropriate dimensions matrices* $N_{1}$, $N_{2}$, *and a scalar* $\alpha>0$, *such that the following inequalities hold* ($i=1,2,\ldots,N$):

17 $$\begin{aligned} \begin{aligned}[b] \Phi_{i}&= \left [ \textstyle\begin{array}{@{}c@{\quad}c@{\quad}c@{\quad}c@{\quad}c@{\quad}c@{\quad }c@{\quad}c@{\quad}c@{\quad}c@{\quad}c@{\quad}c@{}} \Phi_{11i} & \Phi_{12i} & \Phi_{13i} & 0 & \Phi_{15i} & \Phi_{16i} & 0 & \Phi_{18i} & \Phi_{19i} & 0 & \Phi_{1\ 11i} & \Phi_{1\ 12i} \\ * & \Phi_{22i} & 0 & 0 & 0 & 0 & 0 & 0 & 0 & 0 & \Phi_{2\ 11i} & \Phi _{2\ 12i} \\ * & * & \Phi_{33i} & \Phi_{34i} & \Phi_{35i} & 0 & \Phi_{37i} & \Phi _{38i} & 0 & \Phi_{3,10i} & 0 & 0 \\ * & * & * & \Phi_{44i} & 0 & 0 & \Phi_{47i} & 0 & 0 & \Phi_{4,10i} & 0 & 0 \\ * & * & * & * & \Phi_{55i} & 0 & 0 & \Phi_{58i} & 0 & 0 & 0 & 0 \\ * & * & * & * & * & \Phi_{66i} & 0 & 0 & \Phi_{69i} & 0 & 0 & 0 \\ * & * & * & * & * & * & \Phi_{77i} & 0 & 0 & \Phi_{7,10i} & 0 & 0 \\ * & * & * & * & * & * & * & \Phi_{88i} & 0 & 0 & 0 & 0 \\ * & * & * & * & * & * & * & * & \Phi_{99i} & 0 & 0 & 0 \\ * & * & * & * & * & * & * & * & * & \Phi_{10\ 10i} & 0 & 0 \\ * & * & * & * & * & * & * & * & * & * & \Phi_{11\ 11i} & \Phi_{11\ 12i} \\ * & * & * & * & * & * & * & * & * & * & * & \Phi_{12\ 12i} \end{array}\displaystyle \right ] \\ &\leq0, \end{aligned} \end{aligned}$$

*where*

$$\begin{aligned}& \begin{aligned} \Phi_{11i} &=\alpha P_{i}+P_{i}(A+A_{i})+(A+A_{i})^{T}P_{i}+R_{1}+M _{1}- 9e^{-\alpha\tau_{m}}Q_{1} - 12e^{-\alpha\tau_{m}}M_{2}- 12e ^{-\alpha\tau_{M}}M_{3} \\ &\quad{} +N_{2}(A+A_{i})+(A+A_{i})^{T}N_{2}^{T} +(A+A_{i})^{T}N_{1}(A+A_{i})+(A+A _{i})^{T}N^{T}_{1}(A+A_{i}), \end{aligned} \\& \Phi_{12i}=P_{i}(D+D_{i})+N_{2}(D+D_{i})+(A+A_{i})^{T}N_{1}(D+D _{i})+(A+A_{i})^{T}N^{T}_{1}(D+D_{i}), \\& \Phi_{13i}=3e^{-\alpha\tau_{m}}Q_{1}, \quad\quad \Phi_{15i}=-24e^{-\alpha \tau_{m}}Q_{1}- 12e^{-\alpha\tau_{m}}M_{2}, \\& \Phi_{16i}=- 12e^{-\alpha\tau_{M}}M_{3}, \quad \quad \Phi_{18i}=60e^{- \alpha\tau_{m}}Q_{1}+48e^{-\alpha\tau_{m}}M_{2}, \\& \Phi_{19i}=48e^{-\alpha\tau_{M}}M_{3}, \quad\quad \Phi_{1\ 11i}=-N_{2}-(A+A _{i})^{T}N^{T}_{1}-(A+A_{i})^{T}N_{1}, \\& \Phi_{1\ 12i}=P_{i}(B+B_{i})+N_{2}(B+B_{i})+(A+A_{i})^{T}N_{1}B+(A+A _{i})^{T}N^{T}_{1}(B+B_{i}), \\& \Phi_{22i}=-(1-\mu)e^{-\alpha\tau_{M}}M_{1}+(D+D_{i})^{T}N_{1}(D+D _{i})+(D+D_{i})^{T}N^{T}_{1}(D+D_{i}), \\& \Phi_{2\ 11i}=-(D+D_{i})^{T}N^{T}_{1}-(D+D_{i})^{T}N_{1},\quad \quad \Phi_{2\ 12i}=(D+D_{i})^{T}N_{1}B+(D+D_{i})^{T}N^{T}_{1}B \\& \Phi_{33i}=e^{-\alpha\tau_{m}}R_{2}-e^{-\alpha\tau_{m}}R_{1} -9e ^{-\alpha\tau_{m}}Q_{1}-9e^{-\alpha\tau_{M}}Q_{2}, \\& \Phi_{34i}=3e^{-\alpha\tau_{M}}Q_{2}, \quad\quad \Phi_{35i}=36e^{- \alpha\tau_{m}}Q_{1}, \quad\quad \Phi_{37i}=-24e^{-\alpha\tau_{M}}Q_{2}, \\& \Phi_{38i}=-60e^{-\alpha\tau_{m}}Q_{1}, \quad\quad \Phi_{3 \ 10i}=60e ^{-\alpha\tau_{M}}Q_{2}, \\& \Phi_{44i}=-e^{-\alpha\tau_{M}}R_{2}-9e^{-\alpha\tau_{M}}Q_{2}, \quad \quad\Phi_{47i}=36e^{-\alpha\tau_{M}}Q_{2},\quad\quad \Phi_{4\ 10i}=-60e ^{-\alpha\tau_{M}}Q_{2}, \\& \Phi_{55i}=-192e^{-\alpha\tau_{m}}Q_{1}- 36e^{-\alpha\tau_{m}}M _{2}, \quad\quad \Phi_{58i}=360e^{-\alpha\tau_{m}}Q_{1}+96e^{-\alpha\tau _{m}}M_{2}, \\& \Phi_{6 6i}=-36e^{-\alpha\tau_{M}}M_{3}, \quad \quad \Phi_{6 9i}=96e^{-\alpha\tau_{M}}M_{3}, \\& \Phi_{77i}=-192e^{-\alpha\tau_{M}}Q_{2},\quad \quad\Phi_{7\ 10i}=360e ^{-\alpha\tau_{M}}Q_{2}, \\& \Phi_{88}=-720e^{-\alpha\tau_{m}}Q_{1}- 288e^{-\alpha\tau_{m}}M _{2}, \\& \Phi_{9 9}=-288e^{-\alpha\tau_{M}}M_{3}, \quad\quad \Phi_{10\ 10i}=-720e ^{-\alpha\tau_{M}}Q_{2}, \\& \Phi_{11\ 11i}=(\tau_{m})^{2}Q_{1}+( \tau_{M}-\tau_{m})^{2}Q_{2}+N _{1}+N^{T}_{1}, \quad \quad \Phi_{11\ 12i}=-N_{1}(B+B_{i})-N^{T}_{1}(B+B _{i}), \\& \Phi_{12\ 12i}=-\frac{\alpha}{w^{2}_{m}}+(B+B_{i})^{T}N_{1}(B+B _{i})+(B+B_{i})^{T}N^{T}_{1}(B+B_{i}). \end{aligned}$$

*Then the reachable sets of system* (1) *are bounded in a ball* $B(0,r)=\{z\in R^{n}|\Vert z\Vert \leq r\}$ *with*

18 $$ r=\frac{1}{\sqrt{\lambda_{\min}(P_{i})}}, \quad i=1,2,\ldots,n. $$

### Proof

In progress in Theorem 1, replacing matrix *A* by $\sum_{i=1}^{N}\theta_{i}(t)(A+A_{i})$, matrix *B* by $\sum_{i=1}^{N} \theta_{i}(t)(B+B_{i})$, matrix *D* by $\sum_{i=1}^{N}\theta_{i}(t)(D+D _{i})$, one can easily get the conclusion. □

### Remark 1

In this paper, the discrete delay $\tau_{m}\leq \tau(t)\leq\tau_{M}$ is of a more general scope than $0\leq\tau(t) \leq\tau$ considered in [1, 27, 28].

### Remark 2

The novel inequalities in Lemma 2 lead to tighter bounds than the Jensen inequality. By means of novel inequalities in Lemma 2, to estimate integral terms in Lyapunov functionals, better bounds for a reachable set are proposed in this paper.

### Remark 3

To compute the smallest bound of a reachable set for linear dynamic systems (1), we solve the optimization problem for a positive scalar $\delta>0$:

19 $$ \begin{aligned} &\min\quad\bar{\delta} \quad\biggl(\bar{ \delta}=\frac{1}{\delta}\biggr) \\ &\text{s.t. }\textstyle\begin{cases} \text{(a)} &P\geq\delta I, \\ \text{(b)} &\text{inequality (2)/(17) in Theorem 1/2}. \end{cases}\displaystyle \end{aligned} $$

### Remark 4

The novel inequalities in Lemma 2 may be used to study the reachable set problem for a linear neutral system, even for a non-linear neutral system.

### Remark 5

In references [2, 21, 22], they used conventional Jensen inequalities $-(h_{2}-h_{1})\int_{t-h_{2}}^{t-h _{1}}z^{T}(s)P z(s)\,ds\leq-(\int_{t-h_{2}}^{t-h_{1}}z(s)\,ds)^{T} P(\int_{t-h_{2}}^{t-h_{1}}z(s)\,ds)$ and $-\frac{1}{2}(h_{2}-h_{1})^{2} \int_{-h_{2}}^{-h_{1}}\int_{t+\theta}^{t-h_{1}}z^{T}(s) Rz(s)\,ds \,d \theta\leq-(\int_{-h_{2}}^{-h_{1}}\int_{t+\theta}^{t-h_{1}}z^{T}(s)\, ds \,d \theta) R (\int_{-h_{2}}^{-h_{1}}\int_{t+\theta}^{t-h_{1}}z(s)\,ds \,d \theta)$ to estimate a single integral and a double integral, respectively. However, in this paper, we use inequalities in Lemma 2.2 to estimate the bound of a single integral and a double integral. Obviously, the bound in this study is more accurate than those in references. Therefore, the conservatism in our work is less than the existing ones.

### Remark 6

It should be noted that if there are more accurate inequalities to estimate the bound of $\int_{a}^{b}z^{T}(s)Rz (s)\,ds$, $\int_{a}^{b}\dot{z}^{T}(s)R\dot{z}(s)\,ds \,d\theta$, $\int_{a}^{b} \int_{\theta}^{b}\dot{z}^{T}(s)R\dot{z}(s)\,ds \,d\theta$, there is still room for further improvement of the proposed results to reduce the conservatism of systems.

### Remark 7

Some literature works researched the stability of second order delay differential equations; see, for example, references [29–33]. In the future, reachable set bounding for second order delay differential equations may be a hot issue, and methods similar to those in this paper may be used to estimate reachable set bounding for second order delay differential equations.

## Examples

In order to compare the obtained results with those in the literature, we provide several numerical examples in the following.

### Example 1

Consider the uncertain time-varying delayed system in [22, 27]:

20 $$ \begin{aligned} &A+A_{1}= \left [ \begin{matrix} -2 & 0 \\ 0 & -0.7 \end{matrix} \right ] , \quad\quad A+A_{2}= \left [ \begin{matrix} -2 & 0 \\ 0 & -1.1 \end{matrix} \right ] , \quad\quad D+D_{1}= \left [ \begin{matrix} -1 & 0 \\ -1 & -0.9 \end{matrix} \right ] , \\ &D+D_{2}= \left [ \begin{matrix} -1 & 0 \\ -1 & -1.1 \end{matrix} \right ] ,\quad\quad B+B_{1}=\left [ \begin{matrix} -0.5 \\ 1 \end{matrix} \right ] =B+B_{2}, \quad\quad w^{T}(t)w(t)\leq1. \end{aligned} $$

We consider two cases for discrete delay $\tau(t)$: $0\leq\tau(t) \leq0.7$, $\dot{\tau}(t)\leq\mu<1$ and $0\leq\tau(t)\leq0.75$, $\dot{\tau}(t)\leq\mu<1$. Let *μ* be different values, we compute *δ̄*’s by using optimization problem (19). The computed *δ̄*’s are listed in Table 1 for the forward case and in Table 2 for the backward case. From Tables 1 and 2, we know that the proposed result in Theorem 2 is tighter than the ones in references [22, 27].

**Table 1:** $\pmb{\bar{\delta}}$ **’s in Example** **1** **for** $\pmb{0\leq\tau(t)\leq0.7}$ **,** $\pmb{\dot{\tau}(t)\leq\mu}$

	***μ***							
	**0**	**0.1**	**0.2**	**0.3**	**0.4**	**0.5**	**0.6**	**0.9**
[22]	2.97	3.30	3.85	4.85	6.93	12.84	53.86	-
[27]	1.89	1.94	2.00	2.08	2.19	2.35	2.60	3.51
Theorem 2	1.38	1.51	1.59	1.63	1.70	1.81	1.94	2.05

**Table 2:** $\pmb{\bar{\delta}}$ **’s in Example** **1** **for** $\pmb{0\leq\tau(t)\leq0.75}$ **,** $\pmb{\dot{\tau}(t)\leq\mu}$

	***μ***							
	**0**	**0.1**	**0.2**	**0.3**	**0.4**	**0.5**	**0.6**	**0.9**
[22]	3.34	3.79	4.53	5.88	8.85	18.36	127.70	-
[27]	2.28	2.35	2.45	2.57	2.68	2.85	4.62	5.57
Theorem 2	1.27	1.32	1.36	1.97	2.09	2.17	2.54	3.22

### Example 2

Consider the following uncertain model in [2, 21, 22] with parameters:

21 $$\begin{aligned} \begin{aligned} &A+A_{1}= \left [ \begin{matrix} 0 & -0.0936 \\ 1 & -0.685 \end{matrix} \right ] , \quad\quad A+A_{2}= \left [ \begin{matrix} 0 & -0.1464 \\ 1 & -0.245 \end{matrix} \right ] , \\ & D+D_{1}= \left [ \begin{matrix} -0.1 & -0.35 \\ 0 & 0.3 \end{matrix} \right ] =D+D_{2}, \\ & B+B_{1}=\left [ \begin{matrix} -1 \\ 1 \end{matrix} \right ] =B+B_{2}, \quad\quad \mu=0, \quad \quad\tau_{m}=0, \quad \quad \tau_{M}=0.1, \quad\quad w ^{T}(t)w(t)\leq1. \end{aligned} \end{aligned}$$

When *μ* are different values, we solve inequalities (19) to get $\bar{\delta}'s$ for $\tau_{m}\leq\tau(t)\leq\tau_{M}$, $\dot{\tau}(t)\leq\mu$ with $\tau_{m}=0$, $\tau_{M}=0.1$. To compare with the results in [2, 21, 22], we list computed results by using Theorem 2 in Table 3. One can see that there are no feasible solutions by employing the methods in [21, 22], and one can see easily that the proposed method has more application area.

**Table 3:** $\pmb{\bar{\delta}}$ **’s in Example** **2** **for** $\pmb{0\leq\tau (t)\leq0.1}$ **,** $\pmb{\dot{\tau}(t)\leq\mu}$

Method	[21]	[22]	[2]	Theorem 2
*δ̄*	-	-	2.8686 × 10^4^	7.0825

### Example 3

Consider the following delayed system (3) with parameters:

22 $$ \dot{z}(t)=\left [ \begin{matrix} -2 & 0 \\ 0 & -0.7 \end{matrix} \right ] z(t) +\left [ \begin{matrix} -1 & 0 \\ -1 & -0.9 \end{matrix} \right ] z \bigl(t-\tau(t)\bigr) +\left [ \begin{matrix} -0.5 \\ 1 \end{matrix} \right ] w(t), $$

and $w^{T}(t)w(t)\leq1$.

By using the method in Theorem 1, we list computed *r*’s for different values of $\tau(t)$ with $\mu=0$ in Table 4. We can see that bounds computed in this paper are tighter than those of references [2–4, 8, 10, 22]. Of course, it decreases the conservatism of systems.

**Table 4:** **Computed** ***r*** **’s of Example** **4** **for different values of** ***τ*** **with** $\pmb{\mu=0}$

	***τ***				
	**0.1**	**0.3**	**0.5**	**0.7**	**0.9**
[8]	$\sqrt{0.83}$	$\sqrt{1.28}$	$\sqrt{1.94}$	$\sqrt {2.90}$	$\sqrt{4.46}$
[4]	$\sqrt{0.74}$	$\sqrt{0.92}$	$\sqrt{1.36}$	$\sqrt {2.30}$	$\sqrt{3.51}$
[10]	$\sqrt{0.68}$	$\sqrt{0.80}$	$\sqrt{0.97}$	$\sqrt {1.64}$	$\sqrt{3.22}$
[2]	$\sqrt{0.66}$	$\sqrt{0.75}$	$\sqrt{0.94}$	$\sqrt {1.61}$	$\sqrt{3.14}$
[3]	$\sqrt{0.66}$	$\sqrt{0.75}$	$\sqrt{0.94}$	$\sqrt {1.61}$	$\sqrt{3.14}$
[22]	$\sqrt{0.66}$	$\sqrt{0.75}$	$\sqrt{0.94}$	$\sqrt {1.61}$	$\sqrt{3.14}$
Theorem 2	$\sqrt{0.57}$	$\sqrt{0.68}$	$\sqrt{0.81}$	$\sqrt {1.43}$	$\sqrt{2.10}$

### Example 4

**Figure 1:**
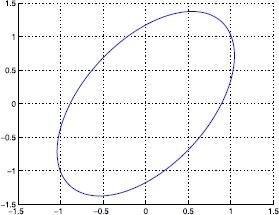
**The reachable set of Example** **4** **.**

Consider the following uncertain delayed system:

23 $$ \begin{aligned} &A+A_{1}= \left [ \begin{matrix} -2 & 0 \\ 0 & -0.7 \end{matrix} \right ] , \quad\quad A+A_{2}= \left [ \begin{matrix} -2 & 0 \\ 0 & -1.1 \end{matrix} \right ] ,\quad\quad D+D_{1}= \left [ \begin{matrix} -1 & 0 \\ -1 & -0.9 \end{matrix} \right ] , \\ &D+D_{2}= \left [ \begin{matrix} -1 & 0 \\ -1 & -1.1 \end{matrix} \right ] , \quad\quad B+B_{1}=\left [ \begin{matrix} -0.5 \\ 1 \end{matrix} \right ] =B+B_{2}, \quad\quad w^{T}(t)w(t)\leq1, \end{aligned} $$

and time delay $0.1\leq\tau(t)\leq0.7$, $\mu=0.7$. The reachable set of system (23) is plotted in Figure 1 with $P=\left [ {\scriptsize\begin{matrix}{} 1.2692 & -0.4984 \cr -0.4984 & 0.7255 \end{matrix}} \right ] $.

### Remark 8

In the reference [29], Domoshnitsky discussed the stability of more complicated linear neutral systems with uncertain coefficients and uncertain delays. In further work, we will study reachable set bounding for this type of linear neutral systems.

## Conclusions

Firstly, we study uncertain linear systems with polytopic parameters. By using L-K functional and novel inequalities to estimate integral terms in L-K functional, some novel sufficient conditions for a bounded reachable set of uncertain systems are obtained. Then, we use some examples to show that our methods in Theorems 1 and 2 are effective and have less conservatism compared with reported conditions. Furthermore, the method in this work may be extended to compute a reachable set of linear neutral systems, and it may even be used to deal with stability of linear systems and non-linear systems in the future.
